# Chronic Ulcerative Stomatitis (CUS) as an Interdisciplinary Diagnostic Challenge: A Literature Review

**DOI:** 10.3390/ijms232213772

**Published:** 2022-11-09

**Authors:** Dominika Cichońska, Dominika Komandera, Magda Mazuś, Aida Kusiak

**Affiliations:** 1 Department of Periodontology and Oral Mucosa Diseases, Medical University of Gdansk, 80-200 Gdansk, Poland; 2 Student Research Group at the Department of Periodontology and Oral Mucosa Diseases, Medical University of Gdansk, 80-200 Gdansk, Poland

**Keywords:** CUS, chronic ulcerative stomatitis, oral mucosa disease

## Abstract

Chronic ulcerative stomatitis (CUS) is a rarely reported disease affecting the oral cavity, most often affecting middle-aged Caucasian females. The aim of the present study is to present the diagnosis, differentiation, and interdisciplinary treatment of this rare disease. CUS is characterized by the presence of an oral erosive or ulcerative lesion. The autoimmune pathogenesis of CUS includes affecting the antigen’s activity by DNA-breaking and protein-hydrolyzing enzymes. The stratified epithelium-specific antinuclear antibodies (SES-ANA) are associated with CUS development. Clinically, the lesions presented in oral mucosa might resemble an erosive form of oral lichen planus, whereas gingival lesions seem to be similar to desquamative gingivitis related to dermatological diseases manifested in the oral cavity. Patients often report subjective symptoms related to oral mucosa and general symptoms. Histopathological presentation of CUS is often non-specific and includes sub-epithelial separation from underlying connective tissue, atrophic epithelium, and inflammatory infiltrate with an increased number of plasma cells and lymphocytes. Direct immunofluorescence (DIF) might be used in CUS diagnostics. CUS generally remains nonsusceptible to corticosteroid treatments; however, antimalarial drugs and calcineurin inhibitors are more effective. Further research should be conducted in order to implement a diagnostic protocol and observe the long-term results of CUS management.

## 1. Introduction

Chronic ulcerative stomatitis (CUS) is a rarely reported disease affecting oral cavities [1]. The first to describe CUS as a distinct entity were Parodi et al. in 1990 [1,2], although, in some reports, preliminary investigative statements were published [3,4,5]. CUS is the disease most often affecting middle-aged Caucasian females, but isolated cases of black and Asian females have also been reported [6]. The majority of the reported cases involved females; however, 10% of CUS cases appeared among male patients [7,8,9]. People aged 35–81 were most often affected by the disease, mainly in the fifth and sixth decades of life [7,8,10]. The average age of the patients diagnosed with CUS was 59 years [7,9,10,11].

CUS is characterized by the presence of oral erosive or ulcerative lesions that display distinctively unique direct and indirect immunofluorescence patterns [7,12]. Not only oral mucosa but also the skin can be affected [6,9]. Presumably, 22.5% of cases involve cutaneous tissues [6]. Chronic ulcerative stomatitis is a debilitating condition, its definition consists of chronic oral ulcers and erosions, which can be surrounded by subtle white reticular striations [1,8]. The clinical similarity of CUS to other oral diseases might be the cause of frequent misdiagnosis and such a small number of described cases [2,10]. After more than 29 years, there are still less than 100 patients reportedly with diagnosed CUS [1]. Azzi et al. highlighted that the mean age of the disease development usually differs from the age of diagnosis. Considering this, the mean diagnostic delay was 30 years, but the most extreme delay was even 30 years, which might be a result of the fact that chronic ulcerative stomatitis still remains a poorly understood disease [1,6,8]. CUS is rarely reported; however this does not enable the consideration of CUS as a rare disease [1].

The aim of the present study is to present the diagnosis, differentiation, and interdisciplinary treatment of this rarely reported disease. Therefore, a descriptive review of the literature on the pathogenesis, diagnosis and treatment strategies has been performed.

## 2. The Autoimmune Pathogenesis

The pathogenesis of chronic ulcerative stomatitis has been investigated since 1990 when Parodi et al. analyzed sera from patients and found circulating antibodies there that targeted a mammalian epithelial antigen. Due to affecting the antigen’s activity by DNA-breaking and protein-hydrolyzing-enzymes, it was assumed that the antigen was a multimolecular, non-histonic DNA-protein complex [1,6]. In the meantime, Jaremko et al. described antinuclear antibodies (ANA) associated with the CUS and identified them as stratified epithelium-specific ANA (SES-ANA) [13]. These antibodies were found in both in vivo and in vitro studies [1,13].

Lee et al. were the first to identify the main antigen which was involved in CUS, a multimolecular 70 kDa epithelial nuclear protein, which they called “chronic ulcerative stomatitis protein” (CUSP) [1,14]. Sequencing the cDNA for CUSP autoantigen revealed that CUSP was homologously similar to the p53 tumor suppressor gene and p73 putative tumor suppressor gene and was a splicing variant of the p53-like rat KET gene [7,14,15]. Therefore, p53 was considered a unique protein for a long time, but in 1997 p63 and p73 were identified as the same family members [16,17]. The p63 gene is located on chromosome 3q26-28 and encodes six p53-homologous proteins [6,16,17]. The structural similarity of the p63 and p73 proteins to the p53 proteins applies to the N-terminal transactivation domain, a central DNA-binding protein, and an oligomerization domain close to the C-terminus [6,16,17,18]. Moreover, each of the p53, p63, and p73 genes use the molecular splicing promoter in different ways, which consequently leads to the formation of various isoforms possessing distinct functional activities. The similarity in the DNA sequences, specifically in the DNA binding domain, enables p53, p63, and p73 to transactivate some of one other’s target genes and to regulate their expression. All of these proteins can interact between themselves [18,19]. The three p53-homologous proteins (TAp63α, TAp63β, and TAp63γ) contain a transactivation domain in N-terminus, such as the p53 protein [6,15,16,17,20]. On the contrary, the other three proteins (∆Np63α, ∆Np63β, and ∆Np63γ) do not have the N-terminal transactivation domain; thus, they are restricted to the epithelium [6,15,17].

Ebrahimi et al. named CUSP as a ΔNp63α protein because it appeared to be an isoform of p63 [1,16]. The p63 exerts a multifaceted effect on embryogenesis, limb morphogenesis, and also the phenotype and development of stratified squamous epithelia, adnexa, teeth, and glands. It is essential for the differentiation, maintenance of proliferative potential, integrity, and apoptotic epithelial injury through the p53 pathway, which may indicate the importance of the presence of the p63 family protein in the oral mucosa due to the constant exfoliation of epithelial cells and a tendency to minor injuries [4,11,18]. ΔNp63α is normally present in the nuclei of the basal and parabasal cells in the progenitor cell compartment of the stratified squamous epithelium and remains the target to autoantibodies with a stratified epithelial specific-antinuclear antibody (SES-ANA) pattern [18,19,20]. ΔNp63α poses an impact on the maintenance of epithelial integrity and homeostasis by regulating the expression of cell-to-cell and cell-to-basement membrane adhesion molecules, which affect epithelial development and regeneration [12,19]. Furthermore, ΔNp63α can block the function of the p53 protein [21].

The very first to describe SES-ANA were Jaremko et al. [13]. These antinuclear antibodies were found circulating in the sera of patients with CUS and were observed only in stratified epithelial substrates and indirect immunofluorescence studies [22]. Furthermore, ANA was not detectable in the muscle or fibrous connective tissue nuclei, conventional ANA substrates, such as the human neoplastic Hep-2 cell line, rodent liver, or kidney substrates. The ANA pattern appears not only on the perilesional mucous membrane but also on normal mucous membranes and skin [7,13]. Solomon et al. described the autoimmune response in CUS patients and showed the presence of IgG antibodies, which are bound to the ∆Np63α antigen. Therefore, among 52% of the patients, circulating IgA antibodies were found [1,6,18]. No positive correlation was observed between patients having both circulating IgG and IgA antibodies and more severe courses of the disease, although patients with mucous membrane pemphigoid and dual circulating IgG and IgA antibodies presented a more severe response to the disease. Future studies are necessary to verify if dual antibodies can also pose an impact on the severity of CUS [6,18].

The highly likely patomechanism of chronic ulcerative stomatitis is that SES-ANA interfere with the normal function of the CUSP protein (∆Np63α) of keratinocytes in the basal and parabasal cell layers of the oral stratified epithelium [4,19]. Due to an intracellular and intranuclear penetration, IgG SES autoantibodies attach to the ΔNp63α of keratinocytes, which cause the detachment from the basal membrane and from each other. Since CUSP is an anti-apoptotic protein, the inhibition of the action of the CUSP by auto-antibody binding leads to apoptotic epithelial injury through the p53 pathway. Clinically, we can observe this as erosions and ulcerations, which are the hallmark of CUS [1,4,10,11,19]. Azzi et al. noted that we do not have a certainty whether the CUS is caused by pathogenic hyperactive IgG autoantibodies binding to ∆Np63α, or if the autoimmune response is handled by physiological IgG reacting to an ∆Np63α overexpression, secondary to the T cell-induced damage to the basal cell layer of the epithelium and to an increase in pro-apoptotic processes [6].

Therefore, some authors have assumed that CUS is not a distinct disease but is recognized as an oral lichen planus variant. This assumption was made due to the fact that autoimmunity directed in ΔNp63α might also be a mechanism involved in LP-epithelial cell damage, which can define CUS as a variant of LP [1,19,23]. Antibodies characteristic of CUS can also be found in LP patients, which was confirmed by Cozzani et al., but some authors claimed that it seems to be an epiphenomenon, which should not be the base of the diagnosis of oral lichen planus (OLP) [1,23]. Contrarily, the other authors believe that chronic ulcerative stomatitis is a hyper-reactive form of OLP, which consists of cytotoxic damage within the basal cells of the epithelium caused by T lymphocytes and the B-cell response to the ∆Np63α antigen. Azzi et al. even proposed a change in the CUS name due to uncertainty if chronic ulcerative stomatitis was a distinct disease [6].

## 3. Clinical Symptoms

The condition generally manifests as non-healing, erosive, or as an ulcerative lesion with subtle white reticular striations [6,13,24]. The most frequent clinical presentations of CUS in the oral cavity are erosions, white lesions, erythema, and ulcerations. Lesions appear on the tongue, which is usually the most common location, followed by the buccal mucosa and the gingiva [7,9,10,12]. The gingival lesions often appear in the form of desquamative gingivitis, which arises from epithelial sloughing due to even minor manipulation of tissue. This can also resemble erosive oral lichen planus (OLP), mucous membrane pemphigoid (MMP), or pemphigus vulgaris, with non-specific lesions or the presence of lichenoid white plaques or striae [6,10,12]. Rarely the hard palate, lingual and labial mucosa, and lower lip might also remain a place of appearance for CUS lesions [7,9,12]. Regardless of its definition, CUS can also be manifested extra-orally, affecting other mucous membranes, skin, hair, and nails [25]. There were also a few reports describing ocular manifestations, such as cicatricial conjunctivitis and ectropion. It was reported that oral lesions can also be accompanied by gluten-dependent enteropathies and genital lesions [6,21].

Regarding the appearance of the oral cavity lesions of CUS, they are mostly symmetrical and might resemble lichenoid lesions, which appear as shallow, irregular ulcerations with abbreviated or vaguely formed peripheral keratotic striae; however, lesions can also present a non-specific clinical picture [6,25,26]. The healing of those lesions does not involve scarring [11]. A lot of authors emphasize that progressive painful erythematous gingival lesions, with large, tender erosions in CUS, which can be indistinguishable from OLP, lichenoid stomatitis, MMP, dermatitis herpetiformis, linear IgA disease, pemphigus vulgaris, erythema multiforme, pyostomatitis vegetans, and epidermolysis bullosa acquisita [7,8,9,11,27,28,29].

Additional reported signs of CUS were the varying severity of desquamation, xerostomia, vesiculation, or positive gingival Nikolsky’s sign. Periodic symptom exacerbations and remissions are often observed [7]. Clinically, the lesions presented in oral mucosa might resemble an erosive form of the oral lichen planus, whereas gingival lesions seem to be similar to desquamative gingivitis related to dermatological diseases manifested in the oral cavity [4,6,30]. Clinical symptoms in CUS are mostly symmetrical and might present an OLP appearance, including white striae departing from the borders of the ulcers observed in 60% of cases [31]. According to conducted research, all patients presented at least one clinically evident CUS symptom, and in most of the cases, more than one clinical sign was observed [3,6,7,10,12,20,25,30,31,32,33,34,35,36,37,38,39]. The lesions’ localization in the oral cavity involved mainly buccal mucosa (nearly 70%) and gingiva (over 50%). Less frequently, lesions were observed on the tongue, hard palate, and labial mucosa. The buccal mucosa and gingiva lesions were more likely to be independently existing lesions. Therefore, lesions in less frequent locations were mostly associated with supplementary lesions in other distinct locations [4,6].

Patients suffering from CUS are affected by painful remitting ulcerations, which are characterized by episodes of remission and exacerbation [8,12]. Patients often report subjective symptoms related to the oral mucosa, such as discomfort or pain and a burning or stinging sensation. General symptoms include nervousness, fatigue, malaise, depression, apathy, and sleeplessness [7,8,11]. Some of the patients reported difficulties in food intake, especially sweet or salty, and drinking hot or cold drinks, which can lead to weight loss in a group of patients [1,7,8]. The inability to maintain proper oral hygiene due to strong pain was also reported by some patients diagnosed with CUS [40].

## 4. Histopathological Presentation

The histopathological presentation of CUS is often non-specific. According to conducted research, over half of histopathological results were classified as “non-specific mucositis”, and nearly half of them were misdiagnosed with lichenoid features [6,41].

The histopathological presentation of CUS was observed as a sub-epithelial separation from the underlying connective tissue, atrophic epithelium, and inflammatory infiltrate with an increased number of plasma cells and lymphocytes. A mixed infiltrate of T-lymphocytes and plasma cells was more specific to chronic ulcerative stomatitis. OLPs’ infiltrate is typically exclusive out of the T-lymphocytes, and its location is limited within a superficial layer of lamina propria [6,24,41,42,43]. Some lesions may present the classic intense, ‘band-like’ inflammatory infiltrate, that is limited to the superficial lamina propria at the interface with the overlying epithelium and a sharply defined deep edge. However, in some CUS cases, a uniform infiltrate extending in some areas into the deeper lamina propria and producing an irregular or hazy deep edge was also observed [6]. The only histological feature that was observed in all CUS cases was the hydropic degeneration of the basal cell layer [6,37]. CUS and erosive oral lichen planus (OLP) manifest in histopathology as an immunological reaction with lichenoid features and a ‘band-like’ inflammatory infiltrate. In CUS predominates, an admixture of T lymphocytes and plasma cells, and in OLP, a predomination of T lymphocytes was observed. However, the lymphocytic infiltrate is not a reliable method for distinguishing CUS from OLP due to the fact that an overlap of the lymphocytic subset is commonly observed and also might be related to other oral mucosa diseases [6,9]. In some CUS cases, the deposition of fibrinogen was reported and described as a fluorescence outlining the basement membrane zone with irregular extensions into the superficial lamina propria, yielding a shaggy appearance. However, further investigation is required to evaluate if a fibrinogen deposition might be perceived as a diagnostic criterion for CUS [6,35].

CUS diagnostics should also include immunofluorescence (IF) microscopy, which is a well-established technique used for the detection of a wide variety of antigens in tissues or on cells in suspension and remains a helpful supplement for the accurate diagnosis of immune-mediated dermatological disorders [44,45].

The direct immunofluorescence (DIF) test for tissue-bound autoantibodies provides a verified adjunct for the diagnosis of dermatological bullous autoimmune disorders, enabling the classification of histologically similar conditions which differ in their treatment protocols and prognosis [45]. In addition, DIF combined with histopathology might complete the clinical and histological examination in the diagnosis of a variety of other dermatological diseases, which include connective tissue disorders, vasculitides, and conditions, such as lichen planus or others. DIF is a one-step procedure that involves the application of fluoresceinated antibodies to a frozen section of the skin or mucous membrane and determines the deposition of the immunoreactants in the tissue [45]. So far, DIF remains a golden standard in CUS diagnosis [6,9]. Reviewed DIF tests yielded a positive result—presenting the characteristic SES-ANA speckled pattern located in the basal layer and the bottom three layers of the cells in nearly all cases [6,46,47]. Among less than half of the reviewed cases, fibrinogen deposition was observed. It was located along the basal membrane zone. In some cases, the adjunctive DIF signals were also observed for the IgA, C3, and IgM components [6,43].

Indirect immunofluorescence (IIF) is a method that requires two incubations and detections of the circulating antibodies in the serum. The patient’s serum is layered on the substrate, followed by the application of fluoresceinated antibodies. An advantage of the IIF is its increased sensitivity (10–15 times). A modified IIF technique using the patient’s own skin as a substrate, known as immunomapping (antigen mapping), is performed to determine the exact site of the cleavage or abnormalities in the distribution of the mutated structural proteins [45]. IIF in CUS diagnosis is the analysis of the SES-ANA autoantibodies located in the basal layer of the epithelium [19]. IIF performed on the remaining negative DIF-analyzed specimens yielded positive. There were also some cases in which DIF has not been performed—the IIF test was always performed on specific epithelial substrates (such as human esophagus/guinea pig, esophagus/monkey esophagus, and esophagus/normal human skin), and all of the IIF test results yielded positive, confirming a CUS presence. Positive IIF results may be used in CUS diagnostics; however, the result is not conclusive, and serum SES-ANA antibodies could also be observed among patients with OLP [6,23,28,48].

The CUS diagnostic protocol might also include an enzyme-linked immunosorbent assay (ELISA) test, which detects the presence of IgG antibodies in the CUS sera. The positive result of an ELISA test for anti-∆Np63α antibodies was observed in patients with clinical symptoms of CUS and played a significant role in distinguishing CUS from other ulcerative diseases and establishing a relationship with OLP [35].

## 5. Diagnostics and Differentiation

The CUS diagnostics should include collaboration between dermatologists, pathologists, and oral clinicians. Among patients with clinically observed long-lasting oral erosions and ulcerations, the DIF analysis should be conducted in order to diagnose CUS [6]. Azzi et al. have proposed a diagnostic criterion for CUS that is presented in Table 1. The major criteria include clinical features, such as chronic painful erosions and/or ulcerations and IgG SES-ANA deposition in the lower third of the epithelium with a speckled pattern in DIF analysis. Minor criteria include CUS symptoms that are often observed; however, these were not reported in all cases and concerning clinical features, histopathology, IIF analysis, laboratory findings, and therapy results. Azzi et al. suggested that for CUS diagnosis, two major criteria should be positive. In cases when DIF analysis is not available, four minor criteria should be observed, including one clinical feature, two among laboratory or histopathological findings, and one therapeutic criterion [6].

Clinical and histological similarities to OLP might be the reason for the misdiagnosis of CUS [6,46]. Oral ulcerations could also be caused by mechanical trauma, oral dysplasia, oral squamous cell carcinoma (OSCC), or hematologic abnormalities [49].

OLP, one of the clinical forms of lichen planus (LP), is a common chronic disorder that generally occurs in patients in the fifth to sixth decades of life and is observed twice more often in females than in males. Oral involvement in LP is very common, and it is assessed that even 15–35% of LP patients might be the only clinical manifestation of the disease. OLP exclusively affects the stratified squamous epithelium that presents as a muco-cutaneous inflammatory disease [42]. Oral mucosa lesions tend to occur as one of three general types: 1. Reticular, including white lines, plaques, and papules which is the most common clinical manifestation; 2. atrophic or erythematous, and 3. erosive, including ulcerations and bullae that resemble CUS lesions [35,50,51]. OLP lesions are mostly symmetrical and are often observed in trauma-prone areas, such as the buccal mucosa and lateral surface of the tongue; however, they might also be present on the gingiva, labial mucosa, and vermilion of the lower lip [2,52]. Erythematous lesions that affect the gingiva cause desquamative gingivitis. Uncommon areas of OLP manifestation are the upper lip, palate, and the floor of the mouth. In the majority of OLP cases, lesions were observed in multiple areas; however, a single patient with lesions isolated to only the lop or tongue has been described. The clinical manifestation of OPL is diversified and might resemble other oral mucosa diseases. Striated white lesions, with or without erosions, might be similar to lupus erythematosus lesions, and the plaque-like OLP lesions may resemble leukoplakia, which is a white keratosis and a precancerous lesion mostly related to tobacco smoking [49,52,53]. The ulcerative form of OLP might be difficult to distinguish from vesiculoerosive dermatological diseases, such as pemphigus and pemphigoid, or can resemble OSCC [49,52]. OLP is a chronic disease that is characterized by periods of exacerbation and remission; however, spontaneous remissions are rarely observed. The pathogenesis of the lichen planus is defined as a lymphocytic immunologic reaction to the epithelial basal cells. The histopathological examination presents basal layer degeneration and apoptotic bodies. In early lesions, the predomination of CD4+ T cells is observed, and in chronic lesions, CD8+ T cells occur more often [35]. A DIF examination in OPL presents a characteristic fibrillar pattern of the fibrin deposition at the basement membrane zone; however, this result in not pathognomonic and can be interpreted only as suggestive OPL. The treatment for OLP can include both local and systemic corticosteroid implementation, calcineurin inhibitors, or retinoids. Taking into consideration only clinical and histopathological presentation, CUS might be indistinguishable from erosive OLP [35,54]. The differentiation of CUS and erosive OLP, including clinical symptoms, histopathology, DIF, and treatment, is presented in Table 2.

Ulcerations in the oral cavity might also result from either acute or chronic trauma. Oral ulcers resulting from acute trauma are generally self-resolving within 14 days without complications; however, chronic ulcerations that are not related to a clear source of trauma require a biopsy to excuse neoplasia or other oral mucosa conditions. The majority of traumatic lesions have nonspecific histologic findings, and treatment should include the removal of the etiologic source of the trauma, promoting healing, and preventing infection [49].

A significant aspect regarding oral ulcers is the diagnosis of ulcerated malignant lesions, such as oral dysplasia and OSCC. All non-healing oral ulcerations require histopathology, especially in a group of patients reporting tobacco and alcohol use. The most malignant suspected are non-symmetrical lesions, and those located on the lateral and ventral surfaces of the tongue and floor of the mouth tend to present a higher risk for malignant transformation. Histopathology depends on the stage of progression and may range from mild to severe dysplasia, carcinoma-in-situ, to invasive carcinoma [49].

There are multiple hematologic abnormalities that may manifest in the oral cavity, including malignant and non-malignant lesions of the B or T-cell origin. Leukemia and neutropenia are the most commonly observed hematological reasons for oral ulcerations. Those conditions also commonly involve gingival bleeding and hypertrophic gingivitis [49,55].

## 6. Treatment Methods

The CUS treatment should promote the healing of erosions and ulcers, relieving the symptoms and preventing secondary infections. CUS generally remains nonsusceptible to both topical and general corticosteroid treatments, on the contrary to other immune-mediated diseases [42,46]. There have been several CUS patient cases treated with corticosteroids—only 11% of them presented therapeutic success (including one combined therapy with dapsone administration). Patients who did not develop a successful response to corticosteroids were passed to antimalarial drugs, mostly chloroquine and hydroxychloroquine combined with corticosteroids or a single drug treatment. Almost half of the treatments resulted in a general improvement or complete clearance, and in over half of the reported cases, this therapy resulted in benefits relapsing when tapering the antimalarial dose [1,8,11]. The improvement or complete healing of the oral lesions was observed after the administration of low doses of hydroxychloroquine (200 mg/day) [35]; however, some authors suggested a higher dosage, even 400 mg/day and 800 mg/day [7]. Hydroxychloroquine interferes with the antigen-processing mechanisms of macrophages and other antigen-presenting cells, which result in the downregulation of the immune response against antigenic peptides. However, the hydroxychloroquine treatment may result in side effects, such as aplastic anemia, agranulocytosis, irreversible retinopathy, toxic psychosis, or neuromyopathy, which leads to the necessity of constant monitoring of patients and a collaboration with the patient’s physician [35]. The therapeutic protocol of CUS may include low doses of antimalarial drugs combined with corticosteroids administrated for a prolonged time [6].

Another approach to CUS management might include tacrolimus, a topical calcineurin inhibitor involved in the production of interleukin-2, which promotes T-lymphocyte proliferation and recruitment. Therefore, it is used in T-cells and mediated diseases, such as eczema, psoriasis, and, potentially, CUS, for its beneficial immunosuppressive effects. According to Stoopler et al., patients undergoing a combined treatment with the antimalarial drug (390 mg hydroxychloroquine) and tacrolimus (0,1%) presented a positive outcome [31]. Cyclosporine, another calcineurin inhibitor, was administered when combined with chloroquine as a CUS treatment method, which also resulted in a successful response [40].

The management of oral lesions among patients with CUS can be challenging, and a multidisciplinary approach is required. The prevention of local irritation by avoiding spicy and hard food with the elimination of alcohol consumption and cessation of smoking plays an essential role. The patient should also be instructed on how to properly care for oral hygiene. It is recommended to use soft toothbrushes and antiseptic mouthwashes, such as chlorhexidine gluconate 0.2%. Topical analgesics, such as benzydamine hydrochloride 0.15% (rinse or spray), might be applied to relieve pain and discomfort, especially prior to eating or tooth brushing. It is also recommended to regularly visit a periodontist in order to remove dental calculus and control periodontal diseases. Sanitation of the oral cavity consisting of the treatment of dental cavities and the removal of non-prognostic teeth allows the elimination of inflammation in the oral cavity. By smoothing the sharp edges of the teeth and parafunction treatment, it is possible to reduce oral injuries that exacerbate CUS-related symptoms. [9,56,57,58,59,60,61].

Summary data on the clinical symptoms, diagnosis, and treatment methods of CUS are presented in Table 3.

## 7. Conclusions

Chronic ulcerative stomatitis (CUS) manifests as non-healing, erosive, or ulcerative lesions in the oral cavity and is an often-misdiagnosed disease due to both its clinical and histological resemblance to other oral mucosa conditions. A proper diagnosis is essential for a successful treatment administration. Further research should be conducted in order to implement a diagnostic protocol and observe the long-term results of CUS management. Taking the presented data into consideration, clinicians should consider the diagnosis of CUS for all erosive or ulcerative lesions appearing cyclically in the oral cavity at the same site, with moderate pain and a slightly specific histopathological picture, after previously excluding traumatic factors.

## Figures and Tables

**Table 1:** Diagnostic criteria for CUS proposed by Azzi et al. [6].

Major Criteria	Minor Criteria
**Clinical features** Chronic painful erosions and/ or ulcerations	**Clinical features** Middle aged or older women Chronic course with relapses Buccal mucosa, tongue (ventral aspect and/or lateral borders), desquamative gingivitis Lichenoid appearance with white striae departing from lesions borders Symetrical distribution Association between diffuse intra-oral distribution and cutaneous lichenoid lesions
**DIF analysis** IgG SES-ANA deposition in the lower third of epithelium with a speckled pattern	**Histopathology** Lichenoid stomatitis, mainly associated with a band-like mixed infiltrate made of lymphocytes and plasma cells
	**IIF analysis** IgG SES-ANA deposition in the basal layer of epithelial substrates with speckled pattern Negative results when using HEp-2 or non-epithelial substrates
	**Laboratory findings** 70 kDa protein detected as autoantigen by immunobinding or other techniques Positive result at ELISA test for anti-∆Np63α antibodies
	**Therapy** Failure or only partial response with corticosteroids Response to hydroxychloroquine (at least 200 mg/day) alone or combined with low doses of corticosteroids

**Table 2:** Differentiation of CUS and erosive OLP.

	Chronic Ulcerative Stomatitis (CUS)	Erosive Oral Lichen Planus (OLP)
**Clinical symptoms**	Oral non-healing ulcerative lesions with subtle white reticular striations located on the tongue, the buccal mucosa, and the gingival tissues (desquamative gingivitis), mostly symmetrical.	Oral mucosa lesions manifested as reticular, including white lines, plaques, and papules; atrophic or erythematous; erosions and ulcerations; mostly symmetrical, located on the buccal mucosa and lateral surface of the tongue, gingiva (desquamative gingivitis) and labial mucosa.
**Histopathology**	Sub-epithelial separation from underlying connective tissue, atrophic epithelium, and inflammatory infiltrate with increased number of plasma cells and lymphocytes (non-specific).	Basal layer degeneration and apoptotic bodies; CD4+ T and CD8+ T cells (non-specific).
**Direct immunofluorescence (DIF)**	SES-ANA speckled pattern, located in the basal layer and the bottom three layers of cells.	Fibrillar pattern of fibrin deposition at the basement membrane zone.
**Treatment**	Chloroquine and hydroxychloroquine combined with corticosteroids or a single drug treatment.	Reticular OLP—observation; erosive OLP—pharmacological treatment (local and systemic corticosteroids implementation, calcineurin inhibitors or retinoids).

**Table 3:** Clinical symptoms, diagnostic tests, and treatment methods of CUS.

	Clinical symptoms	Diagnostic tests	Treatment
Azzi et al. [6]	Painful oral erosions and/or ulcers most often located on the buccal mucosa, the gingiva (desquamative gingivitis), and the tongue.	DIF: SES-ANA deposition, mainly composed of IgGs, in cells of the basal layer and the bottom three layers of cells.	Low doses of antimalarial drugs combined with corticosteroids administrated for a prolonged time.
Islam et al. [7]	Oral erosive or ulcerative lesions located on the tongue, the buccal mucosa, and the gingival tissues (desquamative gingivitis).	DIF: a speckled or finely granular pattern of IgG limited to the basal and parabasal layers of the epithelium, often perinuclear distribution.	Steroidal combination therapy and/or dose regulation of hydroxychloroquine.
Mustafa et al. [9]	Persistent or recurrent painful erosive, ulcerative, vesicular lesions, predominately affecting the tongue, the buccal mucosa, and the gingiva.	DIF: a speckled, finely granular pattern of IgG deposition in the nuclei of keratinocytes. SES-ANA signal is confined to the basal cells and the lower third of the spinous layers.	The same as other oral mucosa erosions and ulcers (no specific treatment was described).
Ko et al. [10]	Oral erosions or ulcerations with periods of exacerbation and remission; the tongue, buccal mucosa, and gingiva are the most commonly affected.	DIF: a speck-led pattern of IgG deposition in the nuclei of keratinocytes limited to the lower layers of the oral squamous epithelium. The presence of SES-ANA distinguishes CUS from oral LP.	No response to corticosteroids.
Solomon et al. [12]	Oral erosive or ulcerative lesions that ale most often present on the tongue, then on the buccal mucosa and gingiva (desquamative gingivitis).	DIF: a speckled, finely granular pattern of IgG deposition in the nuclei of keratinocytes. The SES-ANA signal is confined to the basal cells and lower third of the Malphigian layers.	A combination of small doses of steroids and hydroxychloroquine.
Stoopler et al. [24]	Symptomatic chronic oral ulcers: Wickham’s striae, erythema, and ulceration which commonly affect the buccal mucosa, the tongue, and the gingiva (desquamative gingivitis).	DIF: a speckled pattern of IgG deposition in keratinocyte nuclei limited to the lower layers of the oral squamous epithelium; the presence of SES-ANA antibodies.	Promoting healing, symptom relief, mitigating risks of secondary infection, hydroxychloroquine. No response to corticosteroids.

## Data Availability

Not applicable.
